# The *Herniosina* story continues in the Mediterranean: *H.calabra* sp. nov. from Calabria and *H.erymantha* Roháček, new female from the Peloponnese (Diptera, Sphaeroceridae)

**DOI:** 10.3897/zookeys.1061.72235

**Published:** 2021-10-11

**Authors:** Jindřich Roháček

**Affiliations:** 1 Silesian Museum, Nádražní okruh 31, CZ-746 01 Opava, Czech Republic Silesian Museum Opava Czech Republic

**Keywords:** Biology, distribution, Europe, *Herniosina* Roháček, key, Limosininae, relationships, taxonomy, terminalia

## Abstract

A study of recently acquired material of *Herniosina* Roháček, 1983 (Diptera: Sphaeroceridae: Limosininae) in the Mediterranean subregion revealed a new species, *H.calabra***sp. nov.** (Italy: Calabria: Serre Calabresi Mts) and the first females of *H.erymantha* Roháček, 2016 (Greece: southern Peloponnese: Taygetos Mts). *Herniosinacalabra***sp. nov.** (both sexes) and the female of *H.erymantha* are described and illustrated in detail including structures of terminalia, their relationships are discussed and new information on their biology (habitat association) is given. An update of a key to all know species of *Herniosina* species is presented.

## Introduction

The genus *Herniosina* Roháček, 1983 (Sphaeroceridae: Limosininae) has recently been reviewed by Roháček (2016), including all known species. In the latter study the genus was re-diagnosed without the Nearctic *H.voluminosa* Marshall, 1987 which has been removed so that the genus is monophyletic. *Herniosinavoluminosa* was later transferred to a monobasic genus *Voluminosa* Roháček & Marshall, 2017 because it lacks the majority of the defining apomorphies of the Palaearctic *Herniosina*, see Roháček and Marshall (2017).

*Herniosina* was originally described during the re-classification of the previous genus *Limosina* Macquart, 1835 by Roháček (1982, 1983) for two European species, *H.bequaerti* (Villeneuve, 1917) and *H.horrida* (Roháček, 1978). A third species, *H.pollex* Roháček, 1993, was added by Roháček (1993) from Central Europe and two more species, *H.erymantha* Roháček, 2016 and *H.hamata* Roháček, 2016 have recently been described from the E. Mediterranean area (Greece and Cyprus) by Roháček (2016). In addition, there is a record of an unnamed species of *Herniosina* (based on two females) from Israel (Papp and Roháček 1988: 89, as Herniosinasp.cf.horrida). Discovery of new *Herniosina* species in the eastern Mediterranean (Roháček 2016) indicated that there could also be undescribed species in other parts of the area, and, therefore, our subsequent collecting trips to the Mediterranean have been focused on the acquisition of further material of this largely terricolous and/or subterranean genus. These collecting efforts resulted in two series of *Herniosina* specimens, one from southern Peloponnese (Taygetos Mts) and the other from Calabria (Serre Calabresi Mts). While the former proved to belong to *H.erymantha* and includes the first known females of the species, the other has been recognised to represent an unnamed species morphologically somewhat intermediate between *H.erymantha* and *H.bequaerti*.

The genus *Herniosina* has been newly diagnosed by Roháček (2016: 74–75). This diagnosis does not need a revision after the inclusion of the new species being described below. *Herniosina* species can be most easily recognised by a combination of (largely apomorphic) features in the male abdomen and terminalia (postabdomen strongly down-curved, S1+2 more or less bulging, S5 strongly reduced, cerci modified to peculiar projections, both distiphallus and phallophore projecting posteroventrally) and the (largely plesiomorphic) structures of the female postabdomen (narrow and telescopic, sclerites of 6^th^ and 7^th^ segments and also 8^th^ tergum well developed, no internal sclerites, cerci usually long and slender), however, with reduced S8 and S10.

## Material and methods

### Material

All the material examined (including specimens of *Herniosinabequaerti* used for illustrations but not listed below) is deposited in **SMOC**, Slezské zemské muzeum, Opava, Czech Republic.

### Methods of preparation and study of postabdominal structures

Abdomens of some specimens were detached, cleared by boiling several minutes in 10% solution of potassium hydroxide (KOH) in water, then neutralised in 10% solution of acetic acid (CH_3_COOH) in water, washed in water and subsequently transferred to glycerine. Postabdominal structures were dissected and examined in a drop of glycerine under binocular microscopes (Reichert, Olympus). Detailed examinations of genital structures were performed with a compound microscope (Zeiss Jenaval). After examination, all dissected parts were put into small plastic tubes containing glycerine, sealed with hot forceps and pinned below the respective specimens. Specimens with abdomen removed and terminalia dissected are indicated in the list of material by the abbreviation **genit. prep.**

### Drawing techniques and photography

Legs were drawn on squared paper using a Reichert binocular microscope with an ocular screen. Details of the male and female genitalia were drawn by means of Abbe’s drawing apparatus on a compound microscope (Zeiss Jenaval) at larger magnification (130–500×). Wings were photographed on the compound microscope Olympus BX51 with an attached digital camera (Canon EOS 1200D). Whole adult (dry-mounted) specimens and wings were photographed by means of a digital camera Canon EOS 5D Mark III with a Nikon CFI Plan 10×/0.25NA 10.5 mm WD objective attached to a Canon EF 70–200 mm f/4L USM zoom lens. The specimen photographed by means of the latter equipment was repositioned upwards between each exposure using a Cognisys StackShot Macro Rail and the final photograph was compiled from multiple layers (20–40) using Helicon Focus Pro 7.0.2. The final images were edited in Adobe Photoshop CS6.

### Measurements

Six main characteristics of the new species were measured: body length (measured from anterior margin of head to end of cercus, thus excluding the antenna), index t_2_ : mt_2_ (= ratio of length of mid tibia : length of mid basitarsus), wing length (from wing base to wing tip), wing width (maximum width), C-index (Cs_2_ : Cs_3_) (= ratio of length of 2^nd^ costal sector : length of 3^rd^ costal sector) and index rm\dm-cu : dm-cu (= ratio of length of section between rm and dm-cu on discal cell : length of dm-cu). All type specimens and also all newly collected specimens of *H.erymantha* were measured.

### Presentation of faunistic data

Label data of primary-type specimens are presented strictly verbatim including information on form and colour of all associated labels. Data from paratypes of the new species and also from formerly unpublished non-type specimens are standardised and presented in full. Phenological and other biological information obtained from the material examined and literature are given in the Biology paragraph.

### Morphological terminology

Morphological terminology follows that used for Sphaeroceridae by Roháček (1998) in the Manual of Palaearctic Diptera including terms of the male hypopygium. The “hinge” hypothesis of the origin of the eremoneuran hypopygium, re-discovered and documented by Zatwarnicki (1996), has been accepted and, therefore, the following synonymous terms of the male genitalia (emanating from other hypotheses) need to be listed (terms used first): ejacapodeme = ejaculatory apodeme, epandrium = periandrium, medandrium = intraperiandrial sclerite, phallapodeme = aedeagal apodeme. Morphological terms of the male postabdomen and genitalia are depicted in Figs 3–13, those of the female postabdomen in Figs 17–19. Abbreviations of morphological terms used in text and illustrations are listed below.

### Abbreviations of morphological terms used in text and/or figures

**A_1_** anal vein;

**ac** acrostichal (seta);

**ads** additional (setulae) on frons;

**C** costa;

**ce** cercus;

**Cs_2_**, **Cs_3_** -2^nd^, 3^rd^ costal sector;

**CuA_1_** cubitus;

**dc** dorsocentral (seta);

**dm** discal medial cell;

**dm-cu** discal medial-cubital (= posterior, tp) cross-vein;

**dp** distiphallus;

**ea** ejacapodeme;

**ep** epandrium;

**f_1_**, **f_2_**, **f_3_** fore, mid, hind femur;

**g** genal (seta);

**gs** gonostylus;

**hu** humeral (= postpronotal) (seta);

**hy** hypandrium;

**ifr** interfrontal (seta);

**M** media;

**mt_2_** mid basitarsus;

**oc** ocellar (seta);

**occe** outer occipital (seta);

**occi** inner occipital (seta);

**ors** fronto-orbital (seta);

**pg** postgonite;

**pha** phallapodeme;

**pp** phallophore;

**pvt** postvertical (seta);

**R_1_** 1^st^ branch of radius;

**R_2+3_** 2^nd^ branch of radius;

**R_4+5_** 3^rd^ branch of radius;

**r-m** radial-medial (= anterior, ta) cross-vein;

**S1–S10** abdominal sterna;

**sc** scutellar (seta);

**stpl** sternopleural (= katepisternal) (seta);

**T1–T10** abdominal terga;

**t_1_**, **t_2_**, **t_3_** fore, mid, hind tibia;

**va** ventroapical seta on t_2_;

**vi **vibrissa;

**vte** outer vertical (seta);

**vti** inner vertical (seta).

## Results

### Herniosina calabra sp. nov.

Taxon classificationAnimaliaDipteraSphaeroceridae

4941A937-C08F-5C7C-8122-0F04AE84F135

http://zoobank.org/54BE995C-8C1C-4A16-A968-9EF47B986734

Figs 1 , 2 , 3–7 , 8–13 , 14 , 15–20

#### Type material.

**Figure 1.**
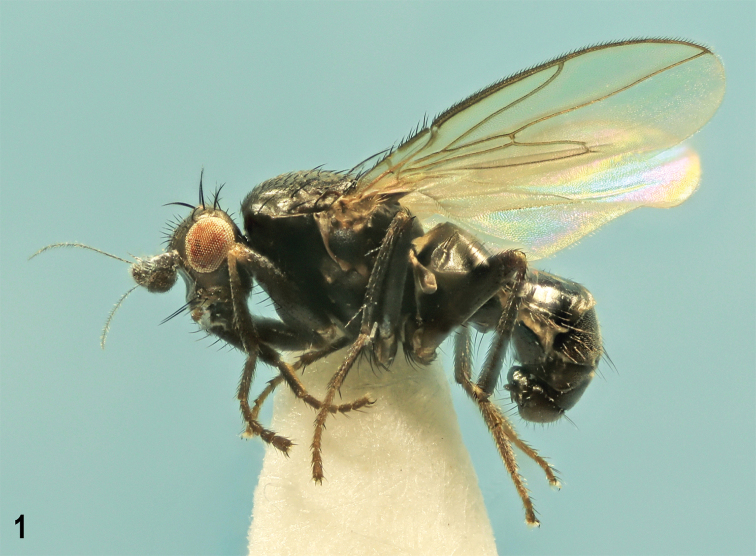
*Herniosinacalabra* sp. nov., male laterally (holotype). Body length ~ 2.3 mm.

***Holotype*** ♂ labelled: “ITALY: W Calabria: Serre Calabresi Mts, Mongiana 2.4 km N, 38°32'05"N, 16°19'06"E “, ”1000 m, 25.5.2018, in tufts of *Juncus* in alder forest, J. Roháček leg.“, ”Holotypus ♂ *Herniosinacalabra* sp. n., J. Roháček det. 2021“ (red label). The specimen is dry-mounted on pinned triangular card, intact (SMOC 06/001/2018-1, Fig. 1). ***Paratypes***: 8♂ 12♀ with same locality labels but with ”Paratypus [♂ or ♀], *Herniosinacalabra* sp. n., J. Roháček det. 2021“ yellow labels; 3♂ 3♀ paratypes with abdomen detached, genitalia dissected and all removed parts preserved in glycerine in coalesced plastic tube pinned below the specimen, 1♂ with wing removed for photography and also preserved in glycerine in pinned plastic tube below the specimen (SMOC 06/001/2018-2 – 06/001/2018-21).

#### Etymology.

The name of the new species is an adjective derived from Calabria (a region in southern Italy) where the type locality of the new species is situated.

#### Description.

**Male** (Fig. 1). Total body length 2.06–2.46 mm; general colour blackish brown with mostly very sparse dark greyish brown microtomentum, hence body relatively shining. ***Head*** blackish brown to brown, lightest on gena. Frons blackish brown posteriorly to brown anteriorly, sparsely microtomentose and largely shining. Occiput blackish brown to black with sparse dark greyish brown microtomentum. Orbits, interfrontalia (very narrow, poorly delimited) and ocellar triangle also greyish brown to dark grey (orbits) microtomentose and duller than rest of frons; frontal triangle relatively narrow, glabrous and shining. Cephalic chaetotaxy: pvt absent, only minute adpressed postocellar setulae behind ocellar triangle; occe distinctly shorter than occi, the latter ~ 2/3 length of vte; vti longest among frontal setae, vte and oc slightly shorter than vti; two strongly exclinate and closely situated ors, posterior longer than anterior and both distinctly shorter than oc; 4 to (usually) 5 relatively short ifr, 1 or 2 middle pairs slightly longer than others; 4 very minute ads inside and below ors; g weak, hardly longer than anterior peristomal seta; vi long, ~ as long as vti. Frontal lunule short, wide, basally brown as anterior margin of frons, apically darkened. Face with cavities below antennae dark brown to black, shining despite sparse greyish microtomentum; medial carina distinct although slightly elevated. Gena high, brown in anterior half, blackish brown posteriorly, sparsely grey microtomentose. Eye relatively small; its longest diameter ~ 1.9 × as long as smallest genal height. Antenna relatively long, black or 3^rd^ segment blackish brown; 3^rd^ segment distinctly tapered apically both in dorsal and lateral view, with cilia on apex as long as those longest on arista. Arista long, ~ 3.8 × as long as antenna, in basal 1/4 short ciliate, otherwise moderately long ciliate.

***Thorax*** dark brown to black, mesonotum relatively shining because of sparse microtomentum, pleuron more densely microtomentose and duller (Fig. 1). Some sutures between pleural sclerites pale brown. Scutellum relatively large and long, rounded triangular, with dorsal surface flat and finely microsculptured, duller than mesonotum. Thoracic chaetotaxy: 2 hu but internal reduced to microseta; 2 postsutural dc, anterior short and weak (only 2 × longer than dc microsetae), posterior strong, ~ as long as or slightly shorter than basal sc; 8–10 rows of ac microsetae on suture; medial prescutellar ac pair somewhat prolonged and thickened but shorter than anterior dc; 2 strong sc, basal slightly longer than scutellum, apical (longest thoracic seta) ~ 1.5 × as long as basal; only 1 stpl because anterior stpl reduced to hardly discernible microseta.

***Legs*** dark brown, coxae, trochanters, knees and tarsi brown to pale brown. f_1_ with sparse and relatively short setae in posterodorsal and posteroventral rows. f_2_ with a row of 4–6 curved but relatively short ventral setae in basal third (Fig. 5) in addition to the usual fine basal seta; t_2_ ventrally with a long row of small dense spines terminated by a strongly reduced va seta (markedly shorter than anteroapical seta), see Fig. 5; dorsal chaetotaxy of t_2_ as in congeners including relatively variable-in-length posterodorsal seta in apical fourth (Fig. 4). t_2_ : mt_2_ = 1.91–2.02.

**Figure 2.**
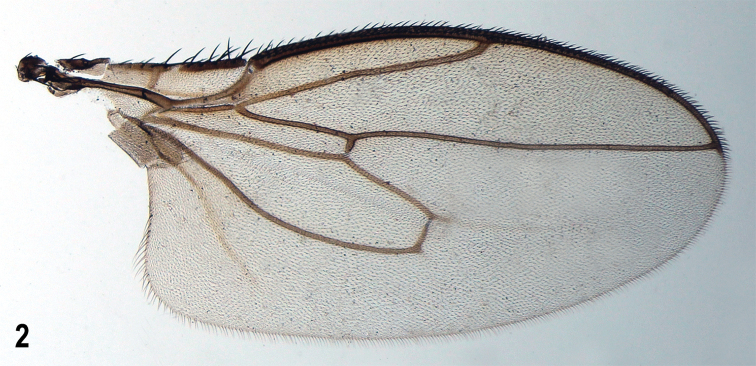
*Herniosinacalabra* sp. nov., wing (male paratype). Wing length ~ 2.1 mm.

***Wing*** (Fig. 2) with pale brownish membrane and pale brown to blackish brown veins. C hardly produced beyond apex of R_4+5_. R_2+3_ slightly sinuate to straight but apically distinctly upcurved to C; R_4+5_ sinuate but with apical half almost straight. Discal cell (dm) variable, relatively short to medium long, distally more or less tapered, usually with small process of M beyond dm-cu (venal fold of M continuing this process usually well visible); posterior outer corner of dm cell obtuse-angled, often with small to minute process of CuA_1_ beyond dm-cu, rarely rounded (1 specimen). A_1_ slightly sinuate; anal lobe well developed; alula narrow but not acute. Wing measurements: length 1.88–2.32 mm, width 0.77–0.97 mm, C-index = 0.87–1.17, rm\dm-cu : dm-cu = 2.87–3.67. Haltere with dirty yellow stem and dark brown knob.

**Figures 3–7.**
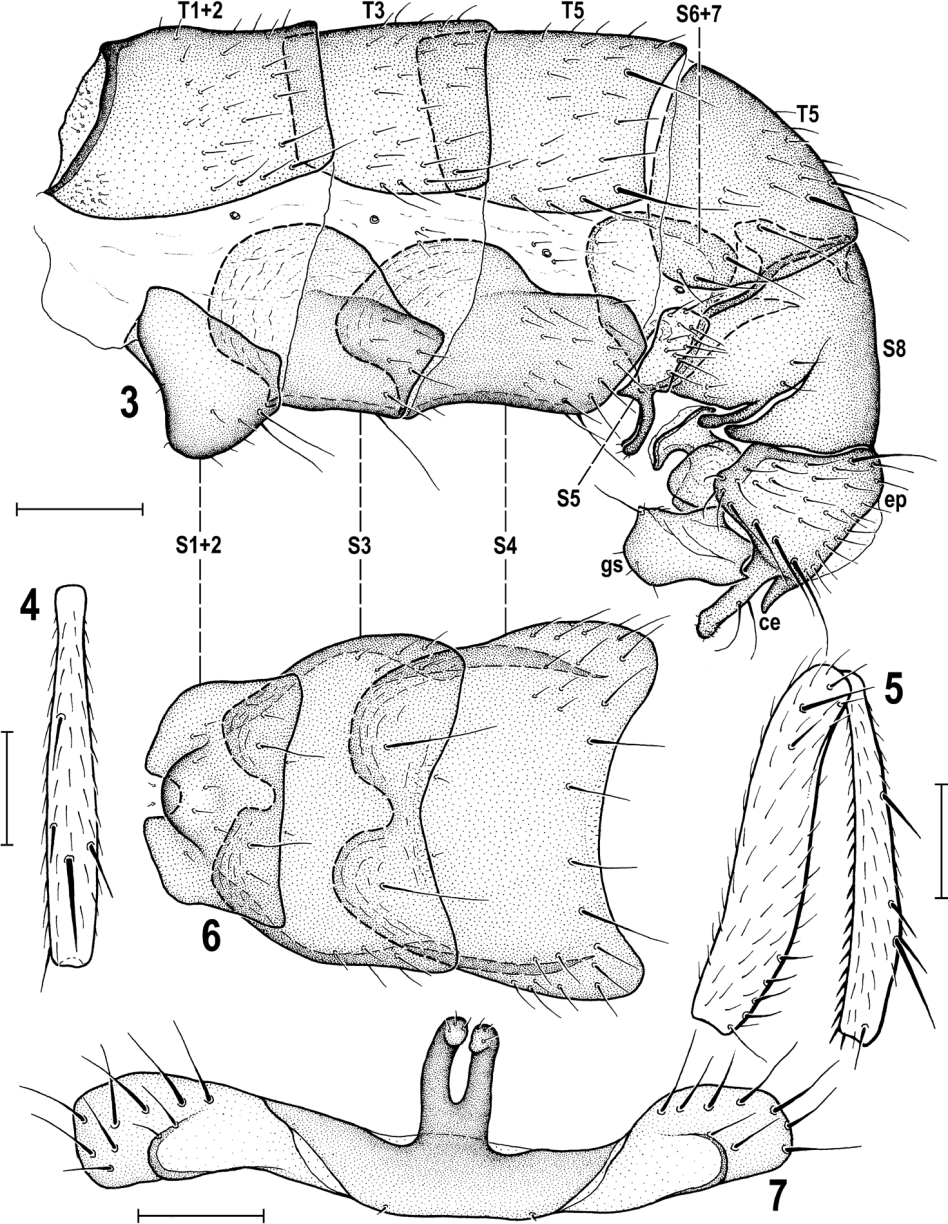
*Herniosinacalabra* sp. nov. (male paratype). **3** Abdomen, laterally **4** mid tibia, dorsally **5** mid femur and tibia, anteriorly **6** preabdominal sterna, ventrally **7** S5, ventrally. Abbreviations: ce – cercus, ep – epandrium, gs – gonostylus, S – sternum, T – tergum. Scale bars: 0.2 mm (**3, 6**); 0.3 mm (**4, 5**); 0.1 mm (**7**).

***Abdomen*** blackish brown to black, with only some postabdominal sclerites brown. Preabdominal terga (Figs 1, 3) large, shining, with only scarce greyish microtomentum, mostly sparsely and shortly setose (but with setae more numerous than in *H.erymantha*). T1+2 longest abdominal tergum. T4 distinctly longer than T3; T5 enlarged, although less than that of *H.bequaerti*, and postabdomen strongly down-curved (Fig. 3). T4 with 1 long seta in each posterior corner; T5 with 4–6 long setae at posterior margin (Fig. 3). Preabdominal sterna modified similarly as in relatives but differing in detail (Figs 3, 6): S1+2 strongly bulging (Fig. 3) and anteromedially narrowly desclerotised, appearing incised (Fig. 6); S3 and S4 deeply anteriorly emarginate due to enlarged posterolateral lobes (Fig. 6); however, these lobes can be smaller (weakly developed) in the smallest specimens; S1+2, S3 and S4 with sparse setae, largely at posterior and lateral margins; S1+2 and S3 with only 1 medial pair of setae long; S4 with 2 pairs of long setae at posterior margin. S5 (Fig. 7) reduced (shortened) and transversely strip-shaped, with pale-pigmented setose lateral parts as in relatives but with darker medial part provided with a long, somewhat flattened (in lateral view slightly bent, see Figs 3, 36) and deeply forked process carrying 2 or 3 setulae on apex of each digitiform lobe (Fig. 7). S6 and S7 coalesced to a complex asymmetrical sclerite hidden under T5 and S8 on left side of postabdomen, narrow ventrally and dorsally but laterally dilated and provided with several flat, keel-like internal lobes (Fig. 3). S8 as long as T5, somewhat tapered posteriorly, with 2 pairs of setulae and with a distinct slit left laterally, the margins of which terminate in 2 slender dark-pigmented digitiform lobes (see Fig. 3).

**Figures 8–13.**
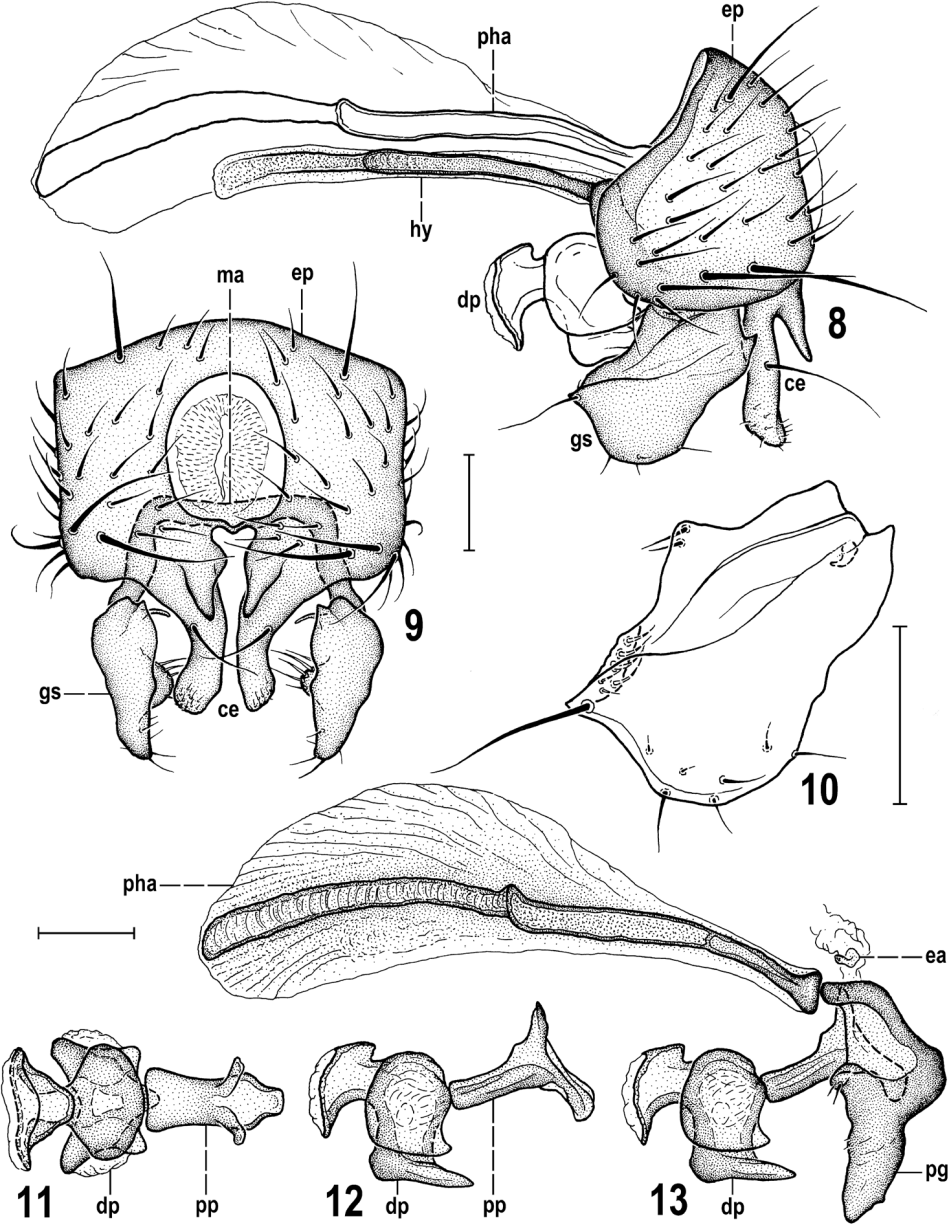
*Herniosinacalabra* sp. nov. (male paratype). **8** Genitalia, laterally **9** external genitalia, caudally **10** gonostylus, laterally **11** aedeagus, dorsally **12** ditto, laterally **13** aedeagal complex, laterally. All scale bars 0.1 mm. Abbreviations: ce – cercus, dp – distiphallus, ea – ejacapodeme, ep – epandrium, gs – gonostylus, hy – hypandrium, ma – medandrium, pg – postgonite, pha – phallapodeme, pp – phallophore.

***Genitalia*.** Epandrium (Figs 8, 9) slightly longer but narrower than that of *H.erymantha* although also angular dorsolaterally (see Fig. 9), with a group of longer and stronger setae laterally and lateroventrally (posterior seta longest and most robust) and also dorsolaterally with 1 longer seta (as in *H.bequaerti*). Anal fissure narrower than high (Fig. 9), suboval, thus more resembling that of *H.bequaerti*. Cerci fused with epandrium, each posteroventrally projecting in 2 processes most similar to those of *H.erymantha*: one (more anterior) robust, almost as long as gonostylus and distally slightly dilated and bearing 1 long seta in addition to series of microsetulae, the other (posterior and more medial) short, lengthwise conical, and bare (Figs 8, 9). Anterior process of cercus differing from that of *H.erymantha* in having distal half distinctly bent out (see Fig. 9). Medandrium low, somewhat reduced and connected by long internal arms with gonostyli (Fig. 9), and posteromedially fused with cerci. Hypandrium with long (though shorter than in *H.bequaerti* and *H.erymantha*) and slender anteromedial rod-like apodeme (Fig. 8). Gonostylus (Figs 8, 9, 10) sub-oblong in lateral view, most resembling that of *H.erymantha* but wider, posterodorsally bearing a distinct tooth (Fig. 10) and its slender dorsal internal process (visible on Fig. 9) short, slightly curved. Aedeagal complex (Figs 11–13) with large and long phallapodeme (as in both relatives) normally provided by large dorsal keel (as in *H.bequaerti*). However, size of phallapodeme and its keel can be reduced in small specimens. Aedeagus most similar to that of *H.bequaerti* because distiphallus is short, with both lateral lobes and an unpaired ventral process short (Figs 11, 12). Postgonite short and robust as that of *H.bequaerti*, differing mainly by robust and non-curved apex (Fig. 13). Phallophore resembling those of both relatives, anteriorly rod-like but dorsoventrally flattened (cf. Figs 11 and 12), posteriorly projecting ventrally and hence epiphallus-like. A minute, pale-pigmented ejacapodeme can be seen close to base of postgonites (Fig. 13).

**Figure 14.**
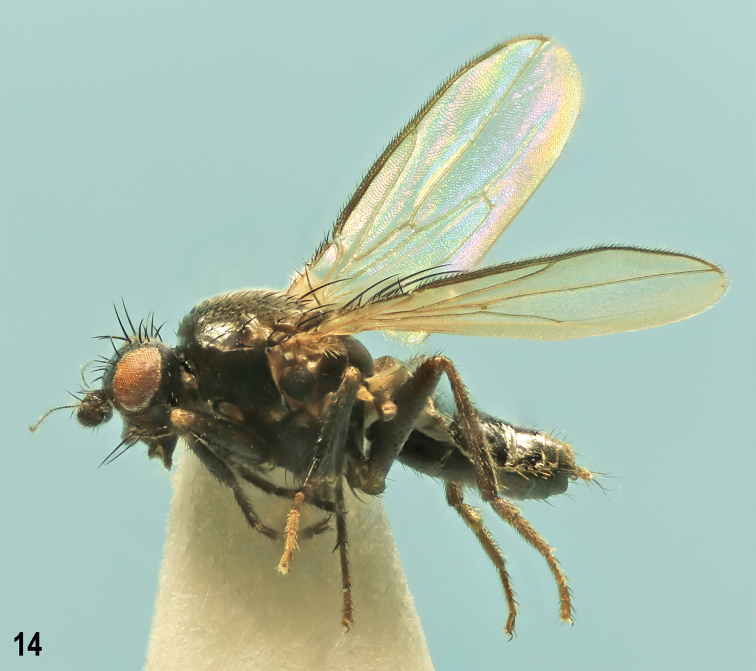
*Herniosinacalabra* sp. nov., female laterally (paratype). Body length ~ 2.2 mm.

**Figures 15–20.**
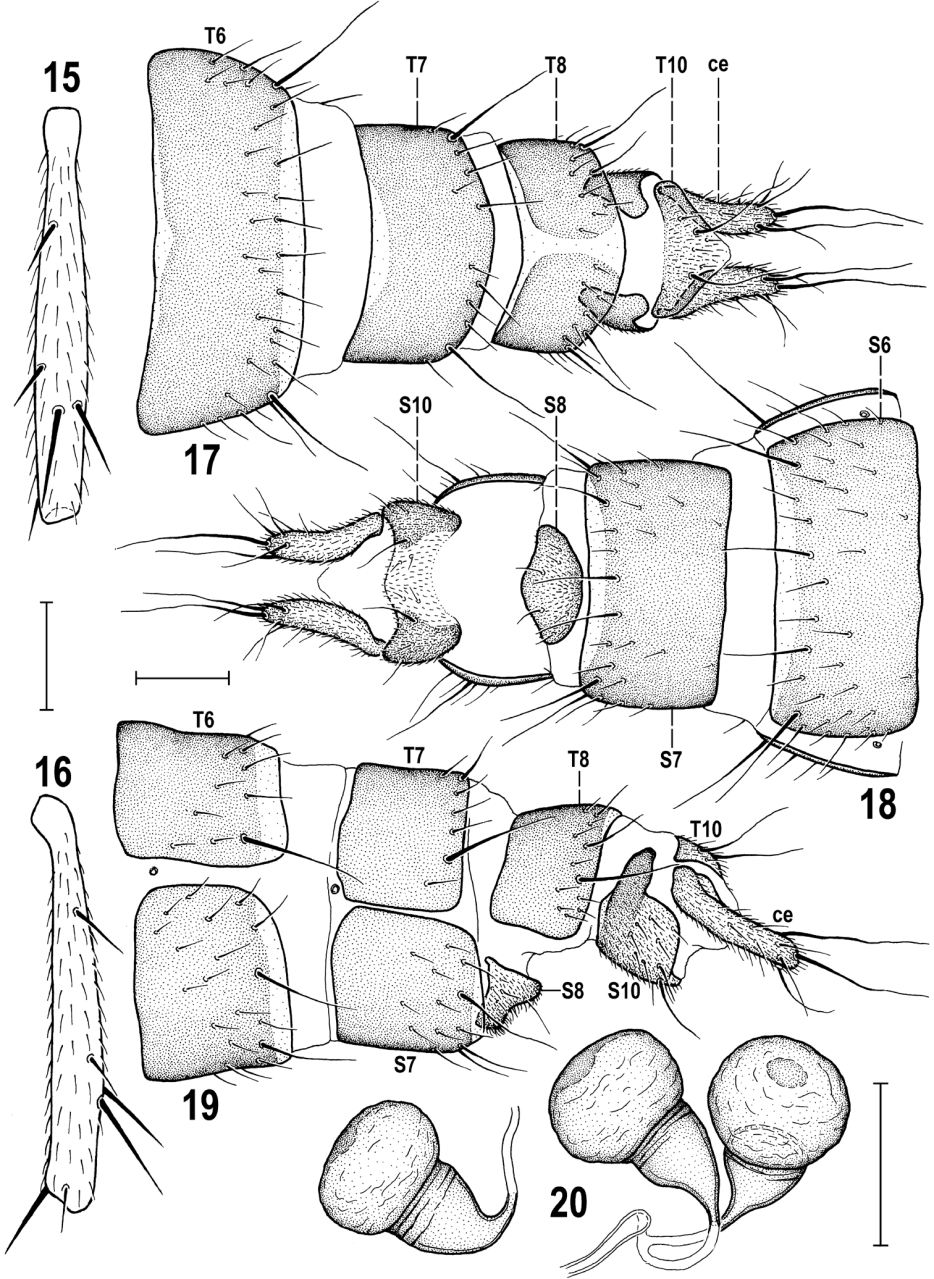
*Herniosinacalabra* sp. nov., female paratypes. **15** Mid tibia, dorsally **16** ditto, anteriorly **17** postabdomen, dorsally **18** ditto, ventrally **19** ditto, laterally **20** spermathecae. Abbreviations: ce – cercus, S – sternum, T – tergum. Scale bars: 0.3 mm (**15, 16**); 0.1 mm (**17–20**).

**Female** (Fig. 14). Similar to male unless mentioned otherwise below. Total body length 2.10–2.78 mm. f_2_ ventrally without curved setae, with only 1 fine basal seta; t_2_ ventrally finely setulose and with 1 long va seta (Fig. 16); anteroapical seta and all setae on dorsal surface of t_2_ somewhat longer (Fig. 15) than in male. t_2_ : mt_2_ = 1.63–1.95. Wing measurements: length 1.83–2.46 mm, width 0.77–1.05 mm, C-index = 0.87–1.06, rm\dm-cu : dm-cu = 2.85–3.75. Preabdominal terga shorter, more transverse and becoming narrower posteriorly, T1+2 widest and longest and with some microtomentum, while T3–T5 almost glabrous and strongly shining; T1+2–T4 similarly setose as in male; T5 unmodified, simply trapezoidal, with setae at posterior margin shorter. Preabdominal sterna unmodified, simple, sparsely and shortly setose and distinctly brownish grey microtomentose, subshiny. S1+2 smallest and dark pigmented only in posterior half; S3–S5 subequal in length but becoming wider posteriorly or S4 as broad as S5; S3 trapezoidal (wider posteriorly); S4 and S5 transversely sub-oblong; all these sclerites blackish brown and shining.

***Postabdomen*** (Figs 17–19) telescopically retractable, basally (6^th^ segment) markedly narrower than preabdomen at 5^th^ segment. 6^th^ segment (both T6 and S6) distinctly wider than 7^th^ segment in contrast to those of *H.bequaerti*. T6 wide and short, transversely trapezoidal, with pale-pigmented anterior and (wider) posterior marginal stripe (Fig. 17), setose at lateral and posterior margins, with longest setae in posterior corners; T7 distinctly narrower than T6 and reaching farther onto lateral side (Fig. 19), with small unpigmented anteromedial area and setosity restricted to posterior margin (Fig. 17). T8 as long as T7 but dorsomedially narrowly depigmented and appearing divided into two dark sclerites (Fig. 17), in contrast to T8 of both *H.bequaerti* and *H.erymantha*. T10 transversely subtriangular (Fig. 17), shorter than those of *H.bequaerti* and *H.erymantha*), pigmented (darkest anterolaterally) except for posterior corner, with a pair of long setae, some fine setulae and micropubescent on almost entire surface. S6 somewhat wider, shorter (more transverse), slightly paler and more setulose than S7 (Fig. 18). S7 dark-pigmented except for posterior marginal stripe and with 4 longer and several short setae at posterior margin. S8 (Figs 18, 19) reduced, short but wider than those of *H.bequaerti* and *H.erymantha*, strikingly convex at anterior margin where densely micropubescent (cf. Fig. 19), otherwise with only 6–8 short setae. S10 reduced to distinctive transverse (in ventral view sinuous) sclerite, being medially depigmented (Fig. 18) but laterally blackish brown and posterodorsally rectangularly incised (Fig. 19), which is also visible in dorsal view (Fig. 17). S10 densely micropubescent and with a few setae including 1 long pair. Spermathecae 2+1 (Fig. 20) blackish brown, pyriform with conical bases, most resembling those of *H.erymantha*, sharing with the latter distally ringed conical bases, dark thickened apex and terminal parts of ducts of paired spermathecae connected rather far from their bodies; however, spermathecae of *H.calabra* are more robust, with wider basal conical parts. Cerci (Figs 17–19) more robust than those of *H.bequaerti* but much longer and narrower than those of *H.erymantha*, each bearing 1 dorsal preapical and 1 apical setae, both very long and sinuate, apart from other shorter setosity and dense micropubescence.

#### Remarks.

*Herniosinacalabra* sp. nov. seems to be morphologically intermediate between *H.bequaerti* and *H.erymantha*. Although seemingly more similar to *H.erymantha* (smaller body size, shorter male T5 and S8, male S5 with deeply forked medial process, anterior process of male cercus long and robust, gonostylus ventrally rounded, not emarginate, spermathecae with conical basal part distally ringed) it is probably most closely related to *H.bequaerti*. Sister-species relationship of *H.calabra* and *H.bequaerti* seems to be particularly demonstrated by the following putative synapomorphies: very similar construction of the male aedeagal complex, including the short distiphallus (with both lateral lobes and unpaired ventral process short) and surprisingly similarly formed, short and robust postgonite. In the female postabdomen there is also a shared synapomorphy: the modified (posterodorsally more or less incised) lateral part of S10 (cf. Fig. 19 and Fig. 42).

The new species can be easily separated from all known congeners only by postabdominal characters. The most species-specific are as follows: the long, slender and deeply forked medial process of male S5 (Fig. 7); the male S8 with digitiform lobes on both sides of lateral slit (Fig. 3); the gonostylus with a posteromedial tooth (Fig. 10), the male cercus with anterior (more lateral) lobe with apex bent outwards (Fig. 9); the postgonite short and with robust apex (Fig. 13); the lateral part of female S10 dark-pigmented and with posterodorsal rectangular incision (Fig. 19). Moreover, the combination of female T8 medially narrowly depigmented with short T10 and relatively long slender cerci (see Fig. 17) is also very characteristic.

#### Biology.

**Figures 21–22.**
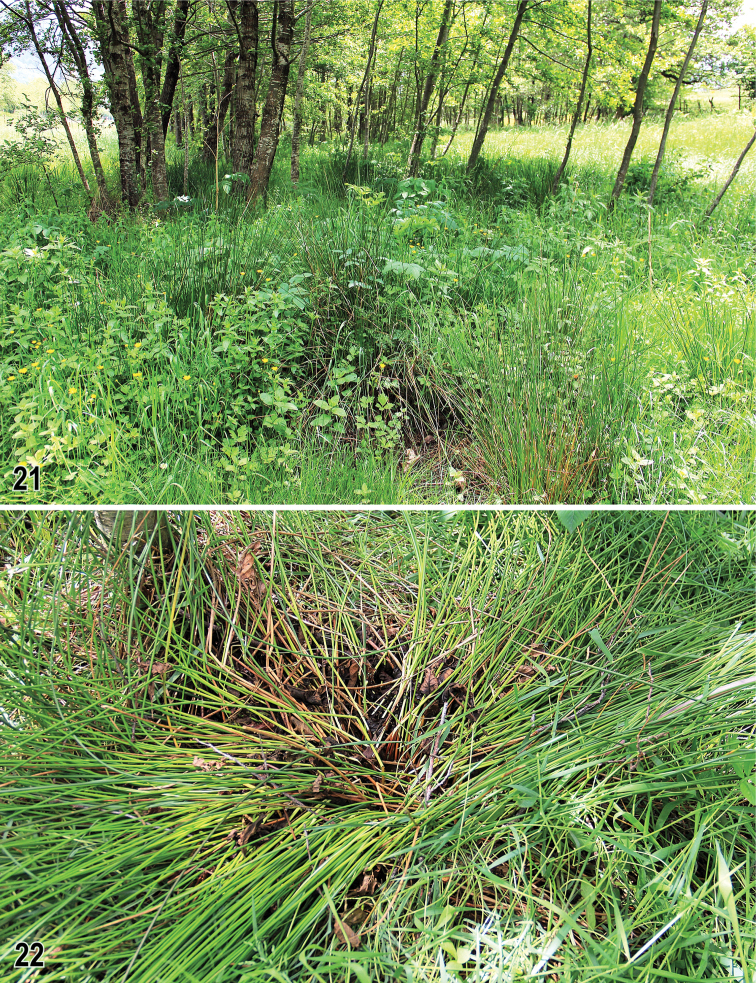
Habitat of *Herniosinacalabra* sp. nov. **21** General view of the habitat in Serre Calabresi at Mongiana, alder trees and herbaceous undergrowth surrounding a small creek (25.v.2018) **22***Juncus* tuft, a microhabitat of the species.

The entire type series of *H.calabra* sp. nov. (21 specimens) was collected (aspirated by a pooter) in May under *Juncus* tufts (Fig. 22) growing under alder trees surrounding a small creek in a montane meadow (Fig. 21). The sphaerocerid community co-occurring with *H.calabra* in and under these tufts of rush (based on collected specimens) proved to be relatively rich and contained the following 15 species: Copromyzinae: *Lotophilaatra* (Meigen, 1830) 2♂3♀, Sphaerocerinae: *Sphaeroceracurvipes* Latreille, 1805 2♂2♀, Limosininae: *Gigalimosinaflaviceps* (Zetterstedt, 1847) 2♂, *Limosinasilvatica* (Meigen, 1830) 5♂3♀, *Opacifronscoxata* (Stenhammar, 1855) 1♀, *Pteremisfenestralis* (Fallén, 1820), 4♂4♀, Pullimosina (Pullimosina) heteroneura (Haliday, 1836) 2♀, P. (P.) pullula (Zetterstedt, 1847) 3♀, P. (P.) vulgesta Roháček, 2001 1♂, *Puncticorpuscribratum* (Villeneuve, 1918) 1♂1♀, *Spelobiaclunipes* (Meigen, 1830) 2♂, *S.palmata* (Richards, 1927) 1♀, *S.talparum* (Richards, 1927) 1♂1♀, S.sp.cf.talis Roháček, 1983 1♂ and *Terrilimosinaschmitzi* (Duda, 1918) 1♀. This assemblage included largely saprophagous terricolous species (such as *H.calabra*, *G.flaviceps*, *Limosinasilvatica*, *Pteremisfenestralis*, *Pullimosina* species, *Puncticorpuscribratum*, *T.schmitzi*) but also a few microcavernicolous species (*Spelobiatalparum*, S.sp.cf.talis) and some ubiquitous, predominantly coprophagous, species (*Lotophilaatra*, *Sphaeroceracurvipes*, *Spelobiaclunipes*). The presence of the latter two groups indicates that there could also be some droppings of small mammals in the detritus. This is for the first time that a species of *Herniosina* has been found under tufts of a graminoid plant. However, rotting leaves of alder were also present under tufts of *Juncus* sp. examined (see Fig. 22), which indicate more resemblance to a leaf-litter association as known in most other *Herniosina* species (cf. Roháček 2016).

#### Distribution.

Hitherto only known from S. Italy (Calabria).

### Herniosina erymantha

Taxon classificationAnimaliaDipteraSphaeroceridae

Roháček, 2016

B3CB7CF2-D709-533F-9333-7F22154C66F0

Figs 23 , 24 , 25–30

Herniosina erymantha Roháček, 2016: 80 [male only, phylogenetic notes, illustr.]. Type locality: Greece, Peloponnese, Alepochori 0.5 km SE.

#### Type material.

***Holotype*** ♂ labelled: ”GREECE: NW Peloponnese: Alepochori 0.5 km SE 37°58'57"N,21°48'10"E“, “590 m, 27.5.2015, sifting leaves under *Platanus*, J. Roháček leg.“, “Holotypus ♂ *Herniosinaerymantha* sp. n., J. Roháček det. 2016“ (red label). The specimen is dry-mounted on pinned triangular card, with left wing and abdomen detached, genitalia dissected and all removed parts preserved in glycerine in coalesced plastic tube pinned below the specimen (SMOC).

#### Other material examined.

Greece: SW Peloponnese: Taygetos Mts, Nedousa 0.5 km W, 37°08'35"N, 22°12'17"E, 390 m, sweeping riverside vegetation, 5.x.2017, 2♂ (1♂ genit. prep.); Taygetos Mts, Artemisia 1 km E, 37°05'47"N, 22°14'27"E, 655 m, sweeping vegetation along brook, 7.x.2017, 1♂, 9.x.2017, 1♀ (genit. prep.); Taygetos Mts, Saidona 1.5 km NE, 36°53'16"N,22°17'59"E, 820 m, sweeping vegetation along brook, 8.x.2017, 1♂ (genit. prep.), all J. Roháček leg. (SMOC).

#### Supplementary description.

**Figure 23.**
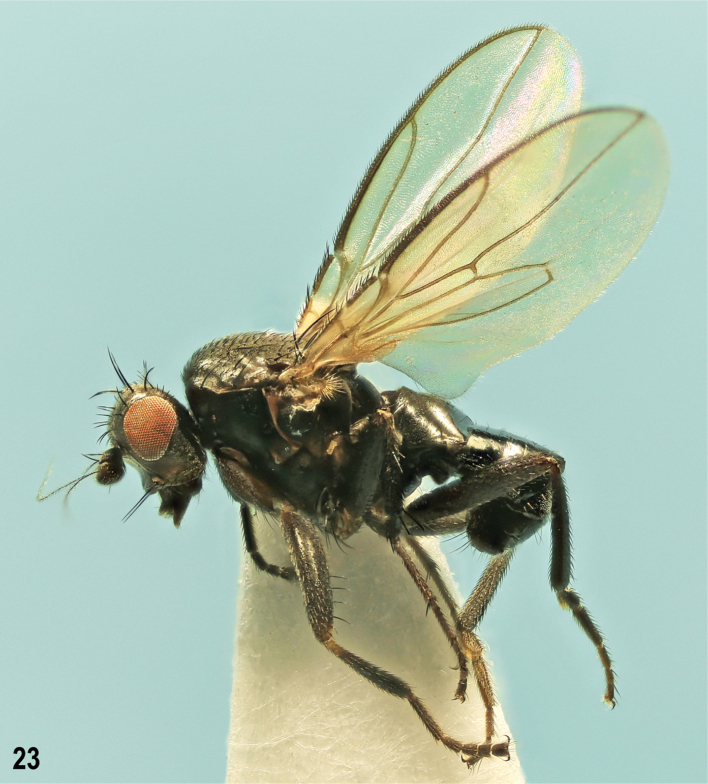
*Herniosinaerymantha* Roháček, 2016, male laterally (Greece: Peloponnese). Body length ~ 2.4 mm.

**Male** (Fig. 23). Total body length 1.79–2.46 mm. ***Head*.** Cephalic chaetotaxy: 3 or 4 relatively short ifr, subequal in length or the middle pair longer. Gena high, usually reddish-brown only anteriorly, sometimes on most of genal surface. Third antennal segment with ciliation on apex as long as longest cilia on arista.

***Thorax*.** Scutellum relatively large and long (1.5 ~ as wide as long), rounded triangular, with dense fine microsculpture on flat dorsal surface. Thoracic chaetotaxy: 1 or 2 stpl, posterior long, anterior reduced to microseta or absent.

***Legs*.** f_2_ with a long row of 6–8 curved but relatively short ventral setae in basal half to two-thirds. t_2_ : mt_2_ = 1.83–1.90.

***Wing*.** Discal cell (dm) variable, relatively short to medium long, distally usually less tapered than in most relatives, with small process of M beyond dm-cu being continued by a venal fold; posterior outer corner of dm obtuse-angled to rounded, sometimes with small remnant of CuA_1_. Wing measurements: length 1.87–2.38 mm, width 0.77–1.01 mm, C-index = 0.88–1.09, rm\dm-cu : dm-cu = 2.62–3.15.

***Abdomen*.** Male S5 (Fig. 37) with medial forked process in lateral view knob-like, distinctly shorter but much more robust (Fig. 38) than that of *H.calabra* (Fig. 36).

***Genitalia*.** Epandrium besides a group of longer and stronger setae laterally and lateroventrally usually also with 1 longer dorsolateral seta which can sometimes be reduced (as is in the holotype, see Roháček 2016: figs 20, 21). Gonostylus (Fig. 41) with posterodorsal corner broadly rounded, never tooth-like and projecting.

**Figure 24.**
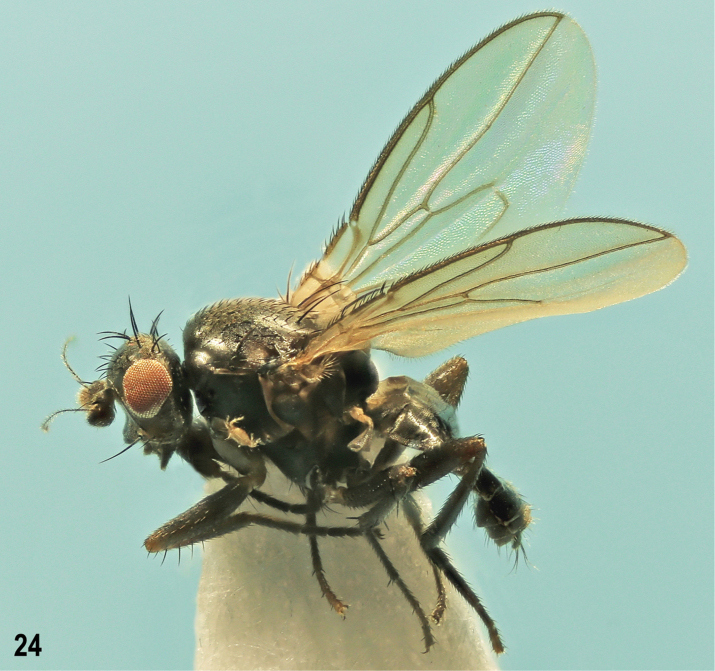
*Herniosinaerymantha* Roháček, 2016, female laterally, with a mite on thoracic pleuron (Greece: Peloponnese). Body length ~ 2.2 mm.

**Figures 25–30.**
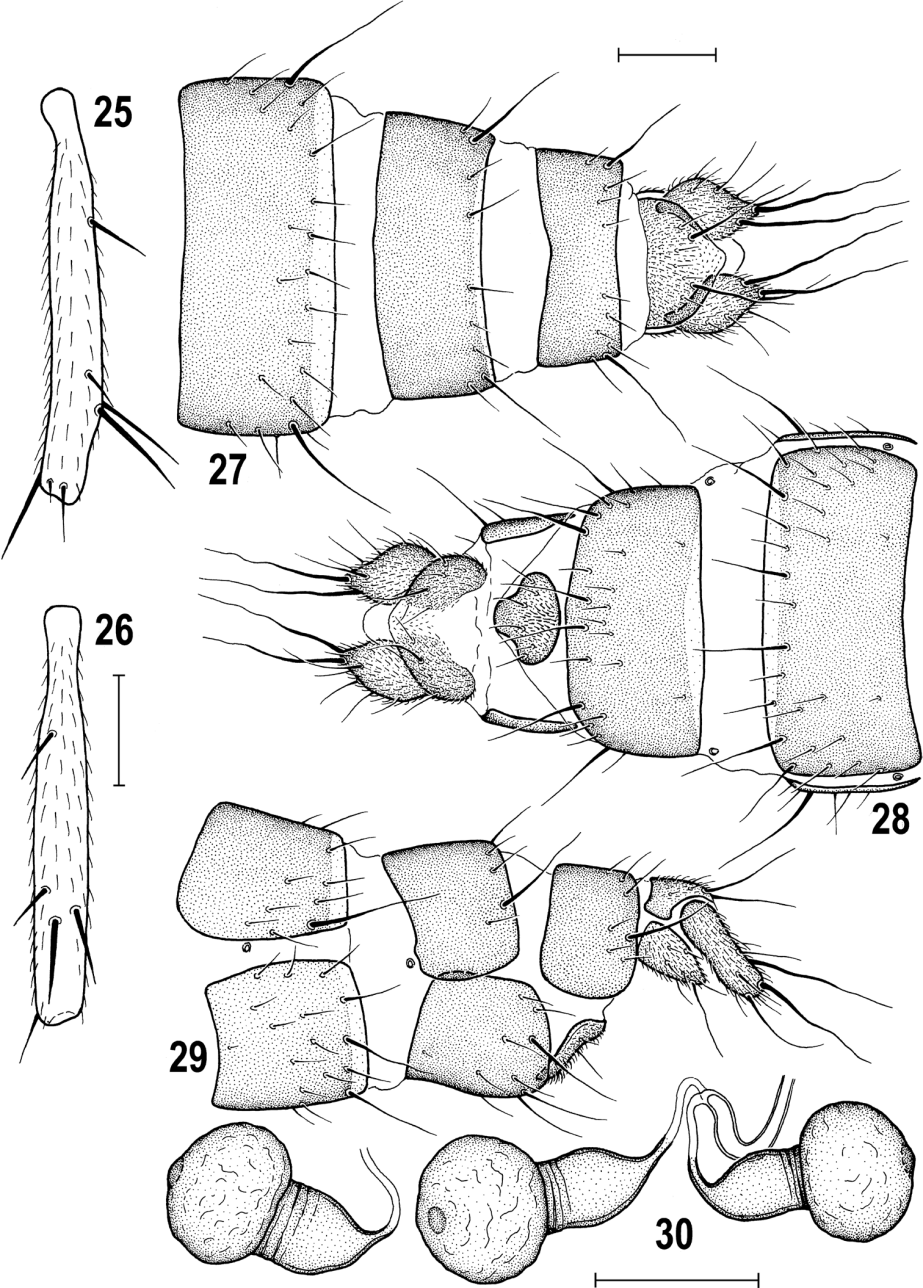
*Herniosinaerymantha* Roháček, 2016, female (Greece: Peloponnese). **25** Mid tibia, anteriorly **26** Ditto, dorsally **27** postabdomen, dorsally **28** ditto, ventrally, **29** ditto, laterally, **30** spermathecae. Scale bars: 0.3 mm (**25, 26**); 0.1 mm (**27–30**).

**Female** (Fig. 24). Similar to male unless mentioned otherwise. Total body length 2.06–2.52 mm. f_2_ ventrally without thicker curved setae, simply setose including 1 long fine basal seta; also t_2_ ventrally finely setulose but with 1 long va seta and anteroapical seta longer than in male (Fig. 25) and longer than in female *C.calabra*; setae on dorsal surface of t_2_ (Fig. 26) also longer than in male, particularly as regards distal posterodorsal seta. t_2_ : mt_2_ = 1.71–1.80. Wing measurements: length 1.91–2.34 mm, width 0.83–0.99 mm, C-index = 0.94–1.07, rm\dm-cu : dm-cu = 2.92–3.46. Preabdominal terga shorter, more transverse and becoming narrower posteriorly, similarly setose as in male. T1+2 widest and longest, covered by sparse but distinct microtomentum apart from posterior marginal stripe; T3–T5 subequal in length, strongly shining because T3 and T4 are glabrous and T5 has microtomentum reduced. Preabdominal sterna unmodified, simple, sparsely and shortly setose and distinctly brownish grey microtomentose, hence less shining than in male. S1+2 smallest, less transverse than S3–S5, darker pigmented only in posterior two-thirds (one fourth to half); S3 and S4 becoming slightly wider posteriorly (S4 largest) and both slightly trapezoidal (wider posteriorly); S5 transversely suboblong, somewhat narrower and shorter than S4; all these sclerites dark brown.

***Postabdomen*** (Figs 27–29) telescopically retractable but broader than in *H.bequaerti* or *H.calabra*, particularly as regards 7^th^ and 8^th^ segments when compared with width of 5^th^ abdominal segment. T6 wide and short, transversely oblong, with pale-pigmented posterior marginal stripe (Fig. 27), sparsely setose at posterior and lateral margin, with 1 long seta in each posterior corner; T7 only slightly narrower than T6 (Fig. 27) but reaching farther onto lateral side (Fig. 29), sparsely setose only at posterior margin and with very narrowly unpigmented posterior margin. T8 shorter and narrower than T7, all dark-pigmented or only narrowly paler at posterior margin medially (Fig. 27). T10 shortly pentagonal, rounded laterally, less transverse than that of *H.calabra* but shorter than that of *H.bequaerti*, pale-pigmented only anteriorly and laterally, and dorsally with a pair of long setae, a few fine setulae and entirely covered by micropubescence (Fig. 27). S6 wider, more transverse and more densely setulose than S7, dark-pigmented except for posterior margin (Fig. 28), with 4 or 6 long posterior setae. S7 also dark but with narrowly unpigmented anterior margin (Fig. 28) and with 4 long (those in medial pair close to each other) setae in addition to sparse short setae in posterior half. S8 (Figs 28, 29) small, narrower than that of *H.calabra*, having posterior half tapered, with several fine setae (4 longer) and distinctive micropubescence, particularly anteromedially. S10 reduced to short, V-shaped, micropubescent and setose sclerite being medially depigmented to interrupted (Fig. 28), with lateral pigmented parts simple (Fig. 29) in contrast to those of *H.calabra*. Spermathecae 2+1 (Fig. 30) blackish brown, elongate pyriform, most resembling those of *H.calabra* but with basal conical parts narrower. Cerci (Figs 27–29) markedly different from those of both *H.calabra* and *H.bequaerti*, unusually short and robust (more so than in *H.hamata* Roháček, 2016), apically conical and dorsoventrally somewhat flattened, each with 1 dorsal preapical and 1 apical seta long sinuate and 1 ventral preapical seta curved (apart form a number of shorter setae), and with dense micropubescence.

#### Remarks.

*Herniosinaerymantha* sp. nov. has only been known from the male holotype (Roháček 2016). A series of specimens recorded here enabled the description of the male to be supplemented and to add the first description of the female. As mentioned above (see Remarks under *H.calabra*), *H.erymantha* seems to be most closely allied to the sister-pair *H.bequaerti* – *H.calabra*. This relationship can now also be confirmed by the female postabdominal characters, including the similar formation of female S8 and, particularly, by the medially depigmented (to almost interrupted) S10 (cf. Fig. 28).

On the other hand, female *H.erymantha* can be easily distinguished from females of both its relatives (and also from all other congeners) by the unusually robust cerci (Fig. 27) and the detailed shape of S8 and S10 (Fig. 28).

#### Biology.

**Figures 31–32.**
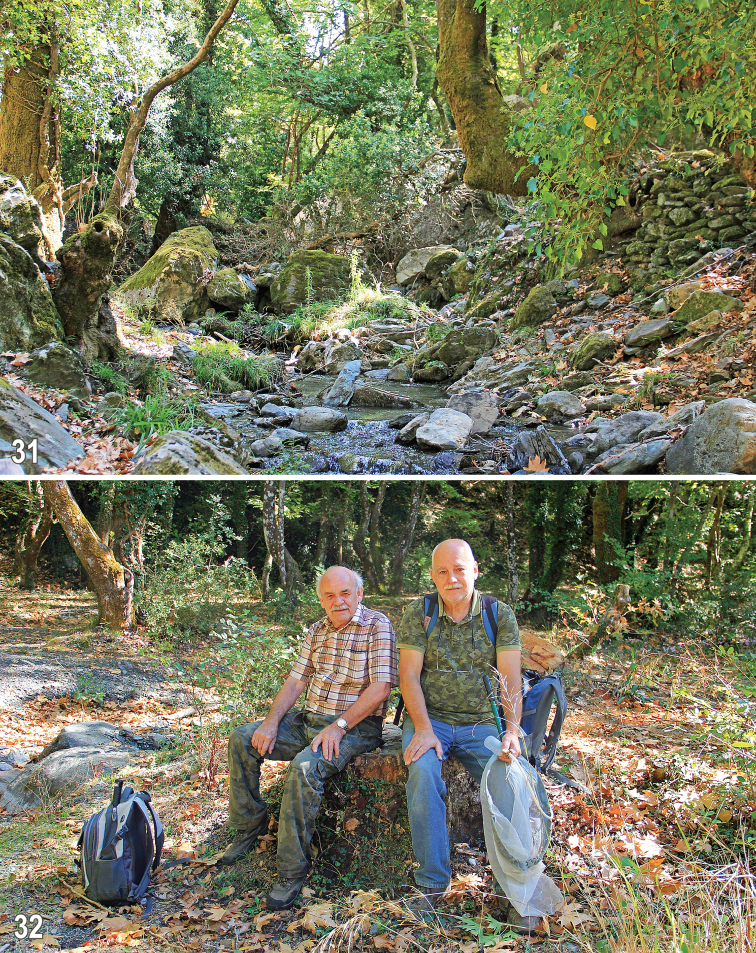
Habitat of *Herniosinaerymantha* Roháček, 2016. **31** valley of montane brook surrounded by *Platanus* trees near Nedousa village in Taygetos Mts (Greece: Peloponnese) (5.x.2017) **32***Platanus* growth near brook near Artemisia village in Taygetos Mts with the Czech dipterists J. Starý (on left) and M. Vála (on right) in foreground (9.x.2017).

Almost all newly obtained specimens of *H.erymantha* were swept from above decaying leaf-litter and sparse vegetation under *Platanus* trees in valleys of montane brooks in the Taygetos Mts (Figs 31, 32), usually mostly in humid places (shores of brooks, springs). Because the holotype was sifted from dead leaves of *Platanus* in a similar montane habitat in the Erimanthos Mts (see Roháček 2016) it is very probable that its larvae develop in this microhabitat. Adults are now known to occur in May (Roháček 2016) and October (present data).

#### Distribution.

Hitherto only known from Greece: Peloponnese.

### An updated key to the identification of *Herniosina* species

**Table:** 

1	Male	**2**
–	Female	**7**
2(1)	S1+2 with a strong protruding bulge (Figs 3, 23).	**3**
–	S1+2 only slightly protruding (Roháček 2016: figs 41, 47, 51).	**6**
3(2)	S5 with a single long medial process that is apically more or less forked (Figs 33, 35, 37); gonostylus in lateral view sub-oblong (Figs 39–41), at most ventrally emarginate (Fig. 39); phallapodeme and hypandrial rod long to very long (Figs 8, 13); posteromedial process of cercus small, with apex more acute (Figs 8, 9); funnel-shaped apex of distiphallus more robust and postgonite with apex simple (Figs 12, 13)	**4**
–	S5 with 2 small digitiform medial processes (Roháček 2016: fig. 36); gonostylus in lateral view with large posteroventral lobe (Roháček 2016: figs 28, 29); both phallapodeme and hypandrial rod short (Roháček 2016: fig. 28); posteromedial process of cercus robust, with apex bluntly rounded (Roháček 2016: figs 28, 30); funnel-shaped apex of distiphallus slender and postgonite with apex curved medially (Roháček 2016: fig. 27).	***H.horrida* (Roháček, 1978)**
4(3)	S5 with medial process long and robust, in lateral view sinuous (Fig. 34), elongate, conical and apically shortly forked (Fig. 33); gonostylus ventrally emarginate (Fig. 39, arrow); cercus with both processes relatively short and both apically pointed (Roháček 2016: figs 4, 5).	***H.bequaerti* (Villeneuve, 1917)**
–	S5 with medial process either short and in lateral view pestle-shaped (Fig. 38) or slender and in lateral view recurved (Fig. 36), always more or less flattened and apically deeply forked (Figs 35, 37); gonostylus ventrally slightly to distinctly rounded (Figs 40, 41); cercus with only medial process short and somewhat pointed; its lateral process very long and robust, apically slightly dilated in lateral view (Roháček 2016: figs 20, 21, Figs 8, 9).	**5**
5(4)	S5 with medial forked process short and robust, pestle-shaped in lateral view (Figs 37, 38); gonostylus without acute posterodorsal corner (Fig. 41) but with long slender curved internal projection (Roháček 2016: fig. 22, Fig. 41 arrow); distiphallus with ventral and lateral lobes relatively long (Roháček 2016: fig. 26) and postgonite longer, slender, with bent but blunt apex (Roháček 2016: fig. 24); f_3_ with long row of 6–8 ventrobasal curved setae (Roháček 2016: fig. 18).	***H.erymantha* Roháček, 2016**
–	S5 with medial forked process long and slender (Fig. 35), digitiform and recurved in lateral view (Fig. 36); gonostylus with acute posterodorsal corner (Figs 10, 40, arrow) and with short internal projection (cf. Figs 9, 40); distiphallus with ventral and lateral lobes short (Fig. 12) and postgonite short, robust (Fig. 13); f_3_ with short row of 4–6 ventrobasal curved setae (Fig. 5)	***H.calabra* sp. nov.**
6(2)	Preabdominal sterna sparsely setose (Roháček 2016: fig. 41); cercus with medial process very long, robust, digitiform and projecting posteroventrally; its lateral process distally slender and laterally provided with a robust long seta arising on small lobe (Roháček 2016: figs 37, 38); gonostylus with lobe-like posteroventral part and internally with a small keel-like process (Roháček 2016: fig. 39); phallophore anteriorly slender, ventromedial lobe of distiphallus simple (unmodified) and postgonite rather straight, with simple apex (Roháček 2016: fig. 40).	***H.pollex* Roháček, 1993**
–	Preabdominal sterna more densely setose (Roháček 2016: fig. 51); cercus without medial process and its lateral process long, slender, apically somewhat dilated, with long seta arising more basally (Roháček 2016: figs 49, 50); gonostylus with a robust posterior internal hook-like process directed ventrally and its posteroventral lobe smaller, knob-like (Roháček 2016: fig. 56); phallophore anteriorly thicker and short, ventromedial lobe of distiphallus projecting far posteriorly and of unusual shape (Roháček 2016: fig. 52) and postgonite proximally dilated and with curved apex (Roháček 2016: fig. 55).	***H.hamata* Roháček, 2016.**
7(1)	T6, T7, S6 and S7 shorter and more transverse (Roháček 2016: figs 58, 62); T8 dorsomedially interrupted into 2 lateral sclerites (Roháček 2016: fig. 58); S8 with membranous window in posterior half (Roháček 2016: figs 62, 64); spermathecae bulbous, without separate basal conical part (Roháček 2016: figs 59, 60).	***H.hamata* Roháček, 2016**
–	T6, T7, S6 and S7 longer, narrower, less transverse (Figs 17, 19, 27, 29); T8 dorsomedially complete (Figs 27, 42), at most medially depigmented (Fig. 17); S8 entirely sclerotised and pigmented (Figs 18, 28); spermathecae pyriform, with distinct basal conical part (Figs 20, 30).	**8**
8(7)	S10 divided into 2 lateral sclerites (Roháček 2016: fig. 8) or medially desclerotised and depigmented (Figs 18, 28); S8 smaller, of various shape (Roháček 2016: fig. 8, Figs 18, 28).	**9**
–	S10 undivided, horseshoe-shaped (Roháček 2016: figs 33, 46); S8 larger, simple, plate-shaped (Roháček 2016: figs 33, 46).	**11**
9(8)	Cerci short and robust (Figs 27, 28); lateral part of S10 simple, posterodorsally without emargination or incision (Fig. 29).	***H.erymantha* Roháček, 2016**
–	Cerci long and slender (Figs 17, 19, 42, 43); lateral part of S10 posterodorsally emarginated to incised (Figs 19, 42).	**10**
10(9)	T6 narrow, hardly wider than T7 (Roháček 2016: fig. 7); T8 dorsomedially simply pigmented, at most with small anteromedial pale-pigmented area (Fig. 43); T10 longer triangular (Fig. 43); lateral parts of S10 posterodorsally slightly emarginated (Figs 42, 43); spermatheca with conical base not ringed distally and its apex with small invagination (Fig. 44)	***H.bequaerti* (Villeneuve, 1917)**
–	T6 broad, wider than T7 (Fig. 17); T8 dorsomedially narrowly depigmented (Fig. 17); T10 short, transversely subtriangular (Fig. 17); lateral parts of S10 posterodorsally with rectangular incision (Fig. 19); spermatheca with conical base distally ringed and its apex with only terminal thickening (Fig. 20).	***H.calabra* sp. nov.**
11(8)	T10 longer, elongately triangular (Roháček 2016: fig. 32); S8 transversely suboval, with only 1 pair of setae (Roháček 2016: fig. 33); cercus longer (Roháček 2016: fig. 34).	***H.horrida* (Roháček, 1978)**
–	T10 shorter, transversely subtriangular (Roháček 2016: fig. 43); S8 more trapezoidal and with 1 pair of longer plus 1–2 pairs of short setae (Roháček 2016: fig. 46); cercus shorter (Roháček 2016: fig. 45)	***H.pollex* Roháček, 1993**

## Discussion

The monophyly of *Herniosina* was demonstrated by Roháček (2016) and its affiliation within the *Limosina* group of genera (sensu Roháček 1982) was confirmed by Roháček and Marshall (2017). Although its closest relationships with *Apteromyia* Vimmer, 1929 (suggested by Roháček 2016: 102) is somewhat questioned by a conflict of the presence of some putative synapomorphies of *Volumosina* Roháček & Marshall, 2017 and *Apteromyia* (cf. Roháček and Marshall 2017: 459), the sister group relationship of *Herniosina* and *Apteromyia* remains as the most probable (Roháček 2016). Particularly, the peculiar modifications of the aedeagal complex (distiphallus with unpaired ventromedial lobe projecting posteriorly; phallophore anteriorly slender and elongate, projecting, movably attached to dorsal side of distiphallus) and the male cerci (formed as robust compact processes below anal fissure) are possible unique synapomorphies of these two genera.

**Figures 33–41.**
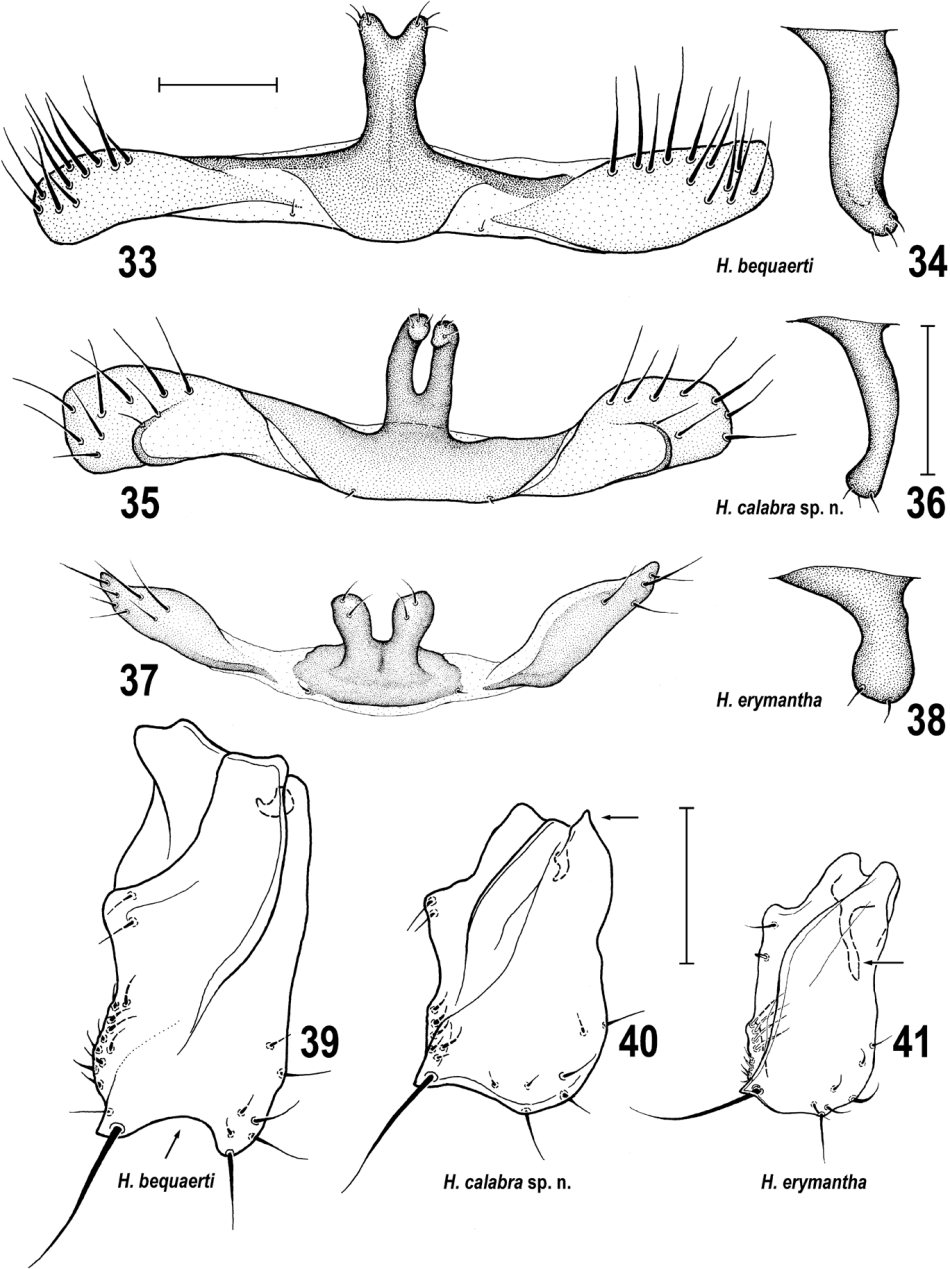
*Herniosina* species, comparison of male characters. **33***H.bequaerti*, S5, ventrally, **34** same species, process of S5, laterally **35***H.calabra* sp. nov., S5, ventrally **36** same species, process of S5, laterally **37***H.erymantha*, S5, ventrally **38** same species, process of S5, laterally **39***H.bequaerti*, gonostylus, laterally **40***H.calabra* sp. nov., gonostylus, laterally **41***H.erymantha*, gonostylus, laterally. All scale bars 0.1 m.

The addition of *Herniosinacalabra* sp. nov. did not affect the concept of the genus which remains a compact group of similar species, differing mainly by the structures of the male and female terminalia. This new species seems to be most closely allied to *H.bequaerti* and *H.erymantha*, and, consequently the hypothesis of the relationships of species within *Herniosina* has to be changed as follows. *Herniosinahamata* is considered a sister-taxon to the five other congeners which belong to a monophyletic group supported by the following putative synapomorphies: male preabdominal sclerites with setosity reduced; male cercus modified to 2 (lateral and medial) processes; gonostylus with dorsal internal projection; spermathecae pyriform, with distinct conical basal part. *Herniosinapollex*, with the bulge of the male S1+2 small (a plesiomorphy shared with *H.hamata*) is considered a sister-group to a clade with *H.horrida*, *H.erymantha*, *H.calabra* and *H.bequaerti* being supported by 2 synapomorphies (male S1+2 strongly bulging; gonostylus with slender dorsal internal projection). *Herniosinahorrida* (having male S5 with a pair of small posteromedial projections = a plesiomorphy shared with *H.hamata* and *H.pollex*) seems to branch off the remaining triplet formed by *H.erymantha*, *H.calabra*, and *H.bequaerti*. The close alliance of these three species is supported not only by 3 synapomorphic features in the male terminalia (S5 with posteromedial distally forked projection; medial process of cercus small and apically pointed; funnel-shaped apex of distiphallus short and robust) but also by female S10 medially desclerotised or divided. Finally, *H.erymantha* is considered a sister-group to *H.calabra* – *H.bequaerti* pair, the latter being supported by similar formation of the aedeagal complex (distiphallus shortened, postgonite short and robust) and modified female S10 having lateral parts posterodorsally emarginated to incised.

**Figures 42–44.**
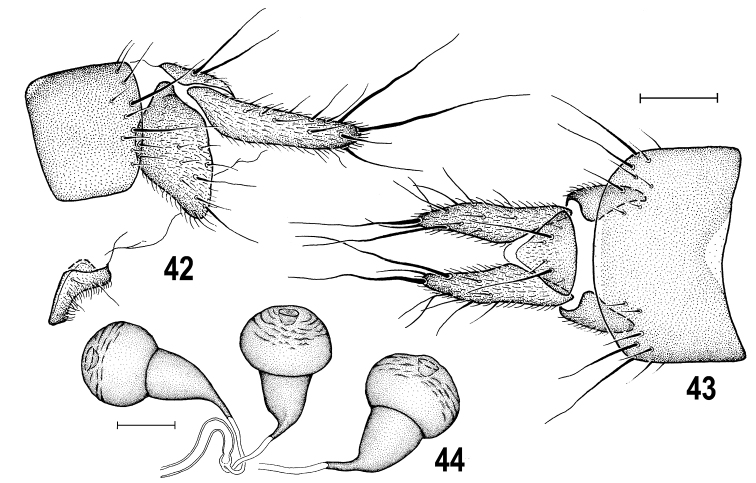
*Herniosinabequaerti* (Villeneuve, 1917), female (Czech Republic: Bohemia). **42** Apex of postabdomen, laterally **43** ditto, dorsally **44** spermathecae. Scale bars: 0.1 mm (**42, 43**); 0.05 mm (**44**).

Discovery of the new species, *H.calabra*, added new information to the general distribution of the genus *Herniosina*. The currently known distribution of the genus ranges from Spain in the west to Russia (Kabardino-Balkariya) in the east and from Iceland and Fennoscandia in the north to Spain, S. Italy, Cyprus and Israel in the south (Roháček et al. 2001; Marshall et al. 2011, present data). The formerly recognised distribution of *Herniosina* in the eastern Mediterranean, viz. in S. Greece (Roháček 2016: *H.erymantha* in northern Peloponnese), Cyprus (Roháček 2016: *H.hamata* in Troodos Mts), and Israel (Papp and Roháček 1988: *Herniosina* sp. in Mt. Hermon) is confirmed by new records of *H.erymantha* from southern Peloponnese (Taygetos Mts) and there is a new occurrence in the middle Mediterranean, viz. from S. Italy (*H.calabra* in Calabria: Serre Calabresi Mts). *Herniosinabequaerti* remains the most widely distributed species occurring throughout Europe (including Iceland) but obviously absent in southeastern parts (cf. Séguy 1963; Roháček 2016). *Herniosinapollex* seems also to be widespread because it is known not only from Central Europe (Czech Republic, Slovakia) but also from the Russian Caucasus (Kabardino-Balkariya), see Roháček (2016). Other known species are probably more restricted. *Herniosinahorrida* is to date only known from central Europe, *H.calabra* from Italy (Calabria), *H.erymantha* from Greece (Peloponnese), *H.hamata* from Cyprus, and *Herniosina* sp. from Israel. Nevertheless, it is probable that these species are in fact more widespread. Because they are terricolous and cavernicolous, all *Herniosina* species are seldom collected by non-specialists, and, therefore, they are poorly represented in the museum collections. This is particularly true for the Mediterranean and other southern parts of the W. Palaearctic region.

## Supplementary Material

XML Treatment for Herniosina calabra

XML Treatment for Herniosina erymantha
